# Rapid and Sensitive Detection of *Plesiomonas shigelloides* by Loop-Mediated Isothermal Amplification of the *hugA* Gene

**DOI:** 10.1371/journal.pone.0041978

**Published:** 2012-10-15

**Authors:** Shuang Meng, Jianguo Xu, Yanwen Xiong, Changyun Ye

**Affiliations:** State Key Laboratory for Infectious Disease Prevention and Control, National Institute for Communicable Disease Control and Prevention, Chinese Center for Disease Control and Prevention, Changping, Beijing, People's Republic of China; Charité-University Medicine Berlin, Germany

## Abstract

*Plesiomonas shigelloides* is one of the causative agents of human gastroenteritis, with increasing number of reports describing such infections in recent years. In this study, the *hugA* gene was chosen as the target to design loop-mediated isothermal amplification (LAMP) assays for the rapid, specific, and sensitive detection of *P. shigelloides*. The performance of the assay with reference plasmids and spiked human stools as samples was evaluated and compared with those of quantitative PCR (qPCR). No false-positive results were observed for the 32 non-*P. shigelloides* strains used to evaluate assay specificity. The limit of detection for *P. shigelloides* was approximately 20 copies per reaction in reference plasmids and 5×10^3^ CFU per gram in spiked human stool, which were more sensitive than the results of qPCR. When applied in human stool samples spiked with 2 low levels of *P. shigelloides*, the LAMP assays achieved accurate detection after 6-h enrichment. In conclusion, the LAMP assay developed in this study is a valuable method for rapid, cost-effective, and simple detection of *P. shigelloides* in basic clinical and field laboratories in the rural areas of China.

## Introduction

*Plesiomonas shigelloides* is a motile, oxidase-positive, facultatively anaerobic, gram-negative rod bacterium, which is presently classified in the family *Vibrionaceae* [1]. *P. shigelloides* has been isolated from a variety of environmental sources, primarily aquatic [2]–[4], and is distributed worldwide. Moreover, *P. shigelloides* has been associated with seafood-associated outbreaks [5]. *P. shigelloides* has been implicated as an agent of human gastroenteritis for many years, with an increasing number of reports describing such infections during the recent years [6]. This bacterium is also of considerable clinical importance as the etiological agent responsible for different types of opportunistic infections [7].

Although extra-intestinal infections such as septicemia, cellulitis, and meningitis caused by *P. shigelloides* are rarely reported, it has been associated with secondary infections in immunocompromised patients [8]–[10]. Salerno et al [12] also described an infection of *P. shigelloides* with a fatal outcome in a newborn. Since most laboratories concentrate on recovery of *Salmonella*, *Shigella*, *E. coli* and other classical enteropathogens, *P. shigelloides* may be overlooked during routine culture of stool samples. The lack of routine analysis for *P. shigelloides* in cases of gastroenteritis leads to only sporadic and occasional identification of this bacterium [11]. However, the greatest challenge to clinicians and epidemiologist is the lack of a rapid, early, and accurate diagnostic method for the detection of *P. shigelloides* as an emerging infectious disease in China.

Several methods such as culture studies and biochemical assays have been developed for detection and identification of *P. shigelloides*. Despite their effectiveness and accuracy, these assays are time consuming, usually requiring up to 5 days to complete. The isolation of *P. shigelloides* from clinical samples has often been unsuccessful owing to the fastidious nature of the organism and the low level of transient bacteremia associated with the disease process. Rapid, specific, and sensitive nucleic acid amplification tests (NAATs) such as standard and real-time PCR have been developed to detect *P. shigelloides* by targeting genes encoding for major virulence factors [5], [13]–[14]. The major limitation to the widespread use of these assays is the fact that a sophisticated thermal cycler is an indispensable requirement of such tests, thereby limiting their wide applicability.

Recently, a novel NAAT technology termed loop-mediated isothermal amplification (LAMP) has attracted a great deal of attention as a rapid, accurate, and cost-effective method for detection of pathogens in clinical diagnostics [15], [16]. LAMP employs 4–6 specially designed primers and a strand-displacing Bst DNA polymerase (isolated from *Bacillus stearothermophilus*) to amplify up to 10^9^ target DNA copies under isothermal conditions (60°C–65°C) within an hour [15], making LAMP a potentially rapid and simple diagnostic tool for detection of *P. shigelloides* infection. In this study, we aimed to develop a rapid, sensitive, and highly specific LAMP assay to detect *P. shigelloides* and evaluate the assay performance with pathogen-simulated human stool.

## Materials and Methods

### Ethics statement

Feces samples were acquired with the written informed consent from a healthy donor. This study was reviewed and approved by the ethics committee of the National Institute for Communicable Disease Control and Prevention, China CDC, according to the medical research regulations of the Ministry of Health, China.

### Bacterial strains and culture conditions

A total of 52 strains (20 *P. shigelloides* and 32 non-*P. shigelloides* strains, as described in Table 1) was used for specificity testing. The bacterial load of the strains used for specificity evaluation was 10^5^ pg/µL, which is high enough to avoid the false-negative amplification. Strain ATCC 51903 was used for the assay optimization , sensitivity evaluation, and simulating human stool samples. *P. shigelloides* and *Enterobacteriaceae* were cultured at 37°C overnight on brain heart infusion agar (BHI; BD Diagnostic Systems, Sparks, MD, USA). Non-*Enterobacteriaceae* strains were grown on blood agar, except for *Vibrio* strains, for which trypticase soy agar (TSA) supplemented with 2% NaCl was used. *Campylobacter* strains were grown under microaerophilic conditions (85% N_2_, 10% CO_2_, and 5% O_2_).

**Table 1:** Strains used in this study.

Latin name	Strain	Strains number
*Plesiomonas shigelloides*	ATCC51903	1
	Isolated strains	19
*Enteropathogenic E. coli*	Isolated strain	1
*Enterotoxigenic E. coli*	Isolated strain	1
*Enteroinvasive E. coli*	Isolated strain	1
*Enterohemorrhagic E. coli*	EDL933	1
*Enteroaggregative E. coli*	Isolated strain	1
*Salmonella enteric*	ATCC14028	1
*Shigella flexneri*	Isolated strain	1
*Shigella sonnei*	ATCC25931	1
*Salmonella typhi*	H98125	1
*Klebsiella pneumoniae*	ATCC700603	1
*Proteus vulgaris*	Isolated strain	1
*Aeromonas veronii*	1.2205	1
*Clostridium perfringens*	Isolated strain	1
*Enterobacter cloacae*	Isolated strain	1
*Serratia marcescens*	Isolated strain	1
*Vibrio parahaemolyticus*	ATCC17802	1
*Staphylococcus aureus*	ATCC6538	1
*Streptococcus pneumoniae*	Isolated strain	1
*Streptococcus pyogenes*	Isolated strain	1
*Streptococcus sanguis*	Isolated strain	1
*Streptococcus salivarius*	Isolated strain	1
*Streptococcus bovis*	Isolated strain	1
*Enterococcus faecalis*	ATCC35667	1
*Yersinia enterocolitica*	ATCC23715	1
*Pseudomonas aeruginosa*	ATCC15442	1
*Aeromonas hydrophila*	ATCC7966	1
*Listeria monocytogenes*	54003	2
*Enterobacter sakazakii*	ATCC51329	1
*Campylobacter jejuni*	ATCC33291	1
*Vibrio minicus*	Isolated strain	1
*Vibrio vulnificus*	Isolated strain	1
*Vibrio fluvialis*	Isolated strain	1

### LAMP primers and reaction conditions

**Figure 1:**
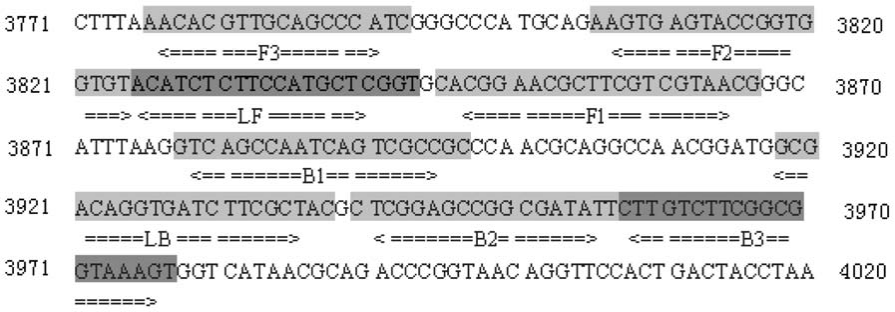
Names and locations of target sequences used as primers for the expression site of *hugA* LAMP.

A set of 6 primers targeted toward the *hugA* gene of the species *P. shigelloides* were designed using PrimerExplorer V4 software (Eiken Chemical Co. Ltd., Tokyo, Japan) based on the conserved sequences determined by the alignment of the *hugA* gene sequences obtained from GenBank. The primers shown in Table 2 were synthesized by Sangon Biotech (Shanghai, China). The primer sequences and their positions in the expression site of the *hugA* gene are shown in Fig. 1. All LAMP reactions were performed with the Loopamp Kit (Eiken Chemical Co. Ltd., Tokyo, Japan) in a 25-µL mixture containing 1.6 µM FIP and BIP primers (each), 0.8 µM LF and LB primers (each), 0.2 µM F3 and B3 primers (each), 20 mM Tris-HCl (pH 8.8), 10 mM KCl, 8 mM MgSO_4_, 10 mM (NH4)_2_SO_4_, 0.1% Tween 20, 0.8 M betaine, 1.4 mM deoxynucleoside triphosphates (dNTPs; each), and 1 µL of Bst DNA polymerase (8 U/µL). The reaction mixture was incubated in a real-time turbidimeter LA320 (Teramecs, Tokyo, Japan) at 65°C for 60 min, followed by 80°C for 5 min to terminate the reaction. Positive and negative samples were distinguished from one another by a turbidity cutoff value of 0.1. After amplification, the LAMP products were detected by electrophoresis on 2% agarose gels with ethidium bromide staining or were determined by visual inspection after adding 1 µL of 1,000× SYBR green I.

**Table 2:** LAMP and qPCR primers used in this study to detect *P. shigelloides.*

Assay type	Primer/Probe name	Sequence (5′-3′)	Position
*hugA*-LAMP	F3	AACACGTTGCAGCCCATC	3776–3973
	B3	ACTTTACCGCCGAAGACAAG	3958–3977
	FIP	CGTTACGACGAAGCGTTCCGTGAAGTGAGTACCGGTGGTGT	3846–3867, 3806–3824
	BIP	GTCAGCCAATCAGTCGCCGCAATATCGCCGGCTCCGAG	3878–3897, 3940–3957
	LF	ACCGAGCATGGAAGAGATGT	3825–3844
	LB	GCGACAGGTGATCTTCGCTAC	3918–3938
*hugA*-qPCR	F	GGAATATCGGCCTGTACAT	4022–4040
	R	TATGGCGGCGATATTTA	4121–4137
	Probe	FAM-CCCCAGACTTTGCTGCGACCATCGG-BHQ-1	4046–4070

### Reference plasmid

To determine the sensitivity of the LAMP assay, a recombinant plasmid containing the target sequence of the *hugA* gene from the *P. shigelloides* strain (ATCC 51903) was constructed as follows: 1) A pair of primers was designed to span the sequences between the F3 and B3 primers; forward primer hugA-F (5′-GCGGTCTCCGGTTTCAAAT-3′) and reverse primer hugA-R (5′-GTTACCGGGTCTGCGTTATG-3′); 2) the PCR products (259 bp) were cloned into the pEASY-T1 vector using the pEASY-T1 Cloning Kit (Transgen, Beijing, China); 3) the recombinant plasmid was quantified with a NanoPhotometer (Implen, Munich, Germany) and was serially diluted (to concentrations of 1×10^6^, 10^5^, 10^4^, 10^3^, 10^2^, 10^1^, and 10^0^ copies/µL) in order to evaluate the limit of detection and the reproducibility of the LAMP assay.

### Evaluation of the sensitivity, specificity, and reproducibility of the LAMP assay

To compare the sensitivities of the LAMP assay and quantitative PCR (qPCR), the serially diluted reference plasmids (at concentrations of 1×10^6^, 10^5^, 10^4^, 10^3^, 10^2^, 10^1^, and 10^0^ copies/µL) containing the target DNA were used to define the limit of detection. The qPCR assay was performed with the primers and probe in Table 2. qPCR amplification was performed in a 20-µL reaction volume containing 0.25 µM primer (each), 0.18 µM probe, 1× Premix (Takara Bio, Inc., Otsu, Japan) Ex Taq™, and 2 µL of DNA template. The assays were conducted using the PCR settings of pre-denaturation at 95°C for 30 s, 40 cycles of denaturation at 94°C for 5 s, and extension at 60°C for 34 s in an ABI PRISM system (Applied Biosystems, Carlsbad, CA, US). Fluorescence readings were acquired using the 6-carboxyfluorescein (FAM) channel.

Genomic DNA of the 32 non-*P. shigelloides* strains were detected by LAMP to determine the specificity of the *hugA* LAMP assay. All detection assays were performed in triplicate.

A set of 3 reference plasmids with varying concentrations (10^6^, 10^4^, and 10^2^ copies/µL) was amplified in two ways (10 times on 1 day and once on each of 10 days) to evaluate the reproducibility of the LAMP assay. The intra-assay coefficient of variation (CVi) and inter-assay coefficient of variation (CVo) were analyzed at the time of peak precipitation, as measured by turbidity on a real-time turbidimeter. Statistical analyses were conducted using SAS software version 9.1.

### LAMP application in simulated human stools

Human stool specimens were obtained from a healthy donor and immediately processed. We have screened the human stool specimens by culture assay and PCR, no *P. shigelloides* strain was detected and no positive amplification of *P. shigelloides* DNA was observed. To determine the detection limit of LAMP in human stool, serial 10-fold dilutions of a mid-log-phase culture of *P. shigelloides* grown in BHI were prepared in phosphate-buffered saline (PBS) and quantified using the standard plating method, resulting in spiking levels between 5×10^7^ and 5×10^1^ CFU/g stool. Aliquots (0.2 g) of the stools were removed for DNA extraction with a QIAamp DNA stool mini kit (QIAGEN, Venlo, Netherlands). This experiment was independently repeated 3 times, and the supernatants (2 µL) were used for both LAMP and qPCR. In addition, the capability of the LAMP assay to detect low levels of *P. shigelloides* in human stool was evaluated. For this application, simulated human stool samples were spiked with *P. shigelloides* cultures at 2 levels: 1 to 2 and 10 to 20 CFU/0.5 g. The samples were homogenized with buffered peptone water (BPW; BD Diagnostic Systems) supplemented with 50 µg/mL ampicillin (Sigma-Aldrich, St. Louis, MO, USA), followed by incubation at 37°C for up to 10 h. Aliquots (1 mL) of the enrichment broth were removed at 4, 6, 8, and 10 h, and processed similarly by QIAamp DNA stool mini kit (QIAGEN). Two microliters of the sample DNA extracts were subjected to both LAMP and qPCR assays, which were repeated twice for each sample.

## Results

### Specificity of the LAMP assay

The specificity of the LAMP assay targeting *hugA* gene was tested with 52 bacterial strains (Table 1). Positive amplifications were observed in 20 *P. shigelloides* strains within a 60-min incubation period. By contrast, 32 non-*P. shigelloides* strains were not amplified after a 60-min incubation period. This result indicates that no false-positive amplifications were observed with these heterologous species in the LAMP assay.

### Sensitivity of LAMP assay

**Figure 2:**
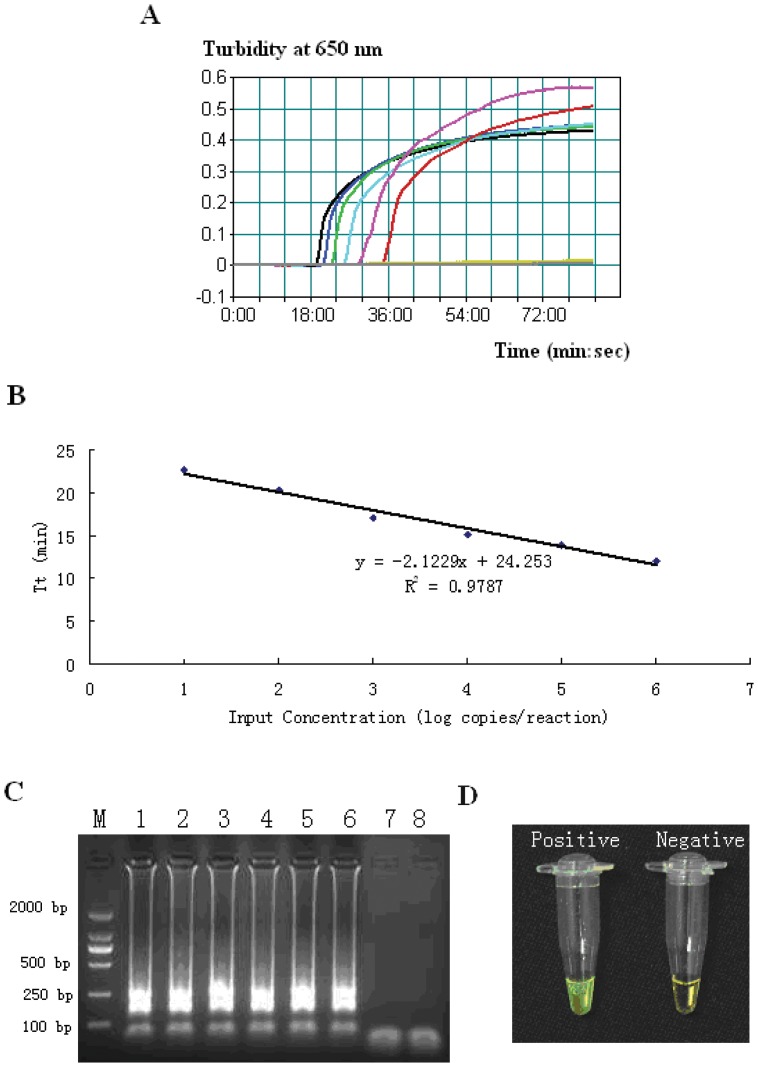
Real-time sensitivity and detection limit of *hugA*-LAMP. (A) Real-time sensitivity of *hugA*-LAMP as monitored by the measurement of turbidity (optimal density at 650 nm). A turbidity of >0.1 was considered to be positive for *hugA*-LAMP. The detection limit was 20 copies/reaction. (B) The relation between the threshold time (Tt) of each sample and the log copies/reaction. The standard curve was drawn on the basis of 3 independent repeats and the linear relationship R^2 = ^0.9787. (C) Sensitivities of electrophoretic analysis of *hugA*-LAMP amplified products. Lane M: DL2000 marker; lane 1: 2×10^6^ copies/reaction; lane 2: 2×10^5^ copies/reaction; lane 3: 2×10^4^ copies/reaction; lane 4: 2×10^3^ copies/reaction; lane 5: 2×10^2^ copies/reaction; lane 6: 2×10^1^ copies/reaction; lane 7: 2×10^0^ copies/reaction; lane 8: no template. (D) SYBR green I fluorescent dye-mediated monitoring of *hugA*-LAMP assay amplification. The original orange color of the SYBR Green I changed to green in case of positive amplification, whereas the original orange color was retained for a negative control with no amplification.

The limit of detection of LAMP (Fig. 2A) and qPCR for the *hugA* gene were 20 and 200 copies/reaction, respectively. This result indicates that the LAMP assay is more sensitive than qPCR for detecting *P. shigelloides* DNA. The LAMP products could also be detected by electrophoresis (Fig. 2C) and visual inspection after adding 1 µL of 1,000× SYBR green I (Fig. 2D).

### Reproducibility of LAMP assay

The CVi was assessed by testing 3 reference plasmids with varying concentrations (10^6^, 10^4^, and 10^2^ copies/µL), 10 times in a single run, whereas the CVo was assessed by testing the same plasmids 10 times in 10 separate runs. The CVi ranged from 1.21% to 1.54%, while the CVo ranged from 2.17% to 3.23%.

### Evaluation of LAMP assay in simulated human stool

**Figure 3:**
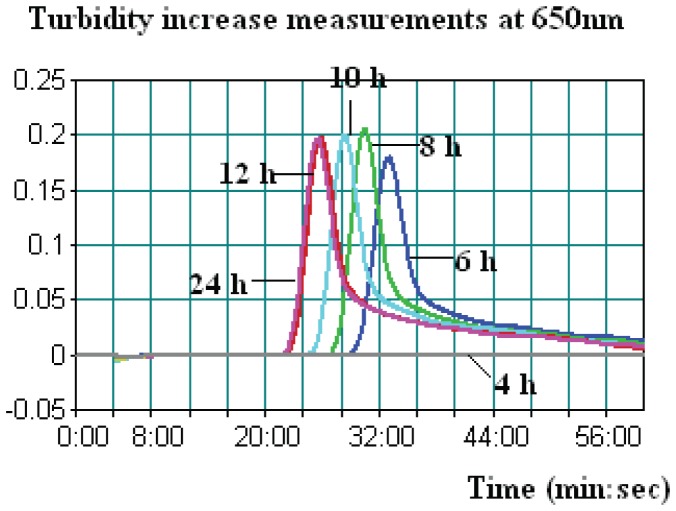
A typical LAMP amplification graph generated when testing human stool samples spiked with the low level of P. shigelloides strain after various enrichment periods (4, 6, 8, 10, 12, and 24 h). In this graph, the human stool sample was spiked with 1.3 CFU of Strain ATCC 51903.

The detection limit of LAMP in simulated human stool was also examined. The LAMP assays detected the presence of *P. shigelloides* strains down to as little as 5×10^3^ CFU/g. By comparison, the qPCR assays had a detection limit of 5×10^4^ CFU/g for *hugA* gene in simulated human stool samples (data not shown). Table 3 summarizes LAMP and qPCR results in human stool samples spiked with 2 low levels (1 to 2 and 10 to 20 CFU/0.5 g) of *P. shigelloides* strains after various enrichment periods. A typical LAMP judgment graph generated for human stool enrichment samples is shown in Fig. 3. Regardless of spiking levels, none of the 4-h-enrichment samples tested positive for *P. shigelloides* by either LAMP or qPCR. We observed positive results with LAMP at 6 h with significantly higher threshold time (Tt) values, while for samples enriched for 8, 10, 12, and 24 h, lower and stable Tt values were observed. A similar trend of detection was observed for qPCR (Table 3). In addition, qPCR results were presented by cycles, which were approximately 1 min/cycle. Therefore, an additional 20–40 min of amplification time was needed for qPCR when testing the same enrichment sample.

**Table 3:** Comparison of LAMP and qPCR assays in human stool samples spiked with low levels of *P. shigelloides.*

Cell level (no. of CFU/0.5 g)	LAMP Tt (min) after enrichment						qPCR CT (cycles) after enrichment					
	4 h	6 h	8 h	10 h	12 h	24 h	4 h	6 h	8 h	10 h	12 h	24 h
1–2	Not available	30.3	27.1	25.2	22.7	22.4	Not available	36.5	33.2	30.8	29.6	29.1
10–20	Not available	28.5	25.3	23.8	21.5	21.3	Not available	33.7	29.1	27.7	26.3	25.8

## Discussion

Some reports have suggested that *P. shigelloides* may cause enteric diseases in normal hosts [5], [17]. Moreover, septicemia, cellulitis, meningitis, and cholecystitis due to *P. shigelloides* have also been documented among immunocompromised patients or patients with other underlying conditions [8]–[10]. The mortality rate associated with *Plesiomonas*-induced septicemia is high [18]. Individuals with serious infections are faced with the lack of a rapid and sensitive diagnostic method and inappropriate antimicrobial therapy, and therefore, they, often, cannot receive timely treatment, leading to diseases and fatal outcomes.

It is well known that the bacteriological methods available for the isolation and identification of *P. shigelloides* are tedious and lengthy. Modified PCR techniques such as nested PCR and real-time PCR are complicated and require a high-precision thermal cycler, and therefore, they are not adapted to diagnosing *P. shigelloides* in basic clinical and field laboratories in rural areas. In contrast, the LAMP assay reported in this study is advantageous because of the following 3 features: rapid reaction, simple operation, and easy detection. The LAMP assay does not require sophisticated and expensive equipment, maintaining a constant temperature of 60°C–65°C for 1 h is sufficient for the reaction [16]. These features demonstrate that the LAMP assay is suitable for the detection of *P. shigelloides* in basic clinical and field laboratories in rural areas.

Although the pathogenesis of *P. shigelloides*-associated gastroenteritis has not yet been elucidated, a number of potential virulence factors have been described [19], [20]. Acquisition of iron is known to be involved in the virulence of a variety of bacterial pathogens [21], [22]. Heme is the most abundant source of iron in the body, and many pathogenic bacteria possess heme transport systems. The *hugA* gene, one of the characterized genes encoded in the heme iron utilization system of *P. shigelloides*, encodes an outer membrane receptor that is required for heme iron utilization.

In this study, all *hugA* gene sequences of *P. shigelloides* recorded in the GeneBank were aligned, and the LAMP primers were designed on the basis of the conserved regions. We tested 32 non-*P. shigelloides* strains to evaluate the specificity of the *hugA* LAMP assay for the bacteria, with the results showing that the specificity of the LAMP assay was 100%.

To the best of our knowledge, this is the first study applying the novel LAMP technology for the detection of *P. shigelloides* in human stool. Previously, spiked samples were usually enriched overnight without characterizing the effects of different enrichment times on the detection outcomes [23], [24]. In this study, *P. shigelloides* strain ATCC 51903 was used in experiments with simulated human stool samples, with the LAMP assays having a detection limit of 5×10^3^ CFU/g stool. Positive detection occurred after a 6-h period of enrichment, and consistently thereafter, for the human stool samples spiked with 2 low levels (1 to 2 and 10 to 20 CFU/0.5 g) of ATCC 51903. We observed that the LAMP assay performed better than qPCR with respect to detection limit and assay speed in spiked human stool. In general, molecular level-based detection methods such as PCR and LAMP are subjected to a variety of inhibitors present in clinical samples. Some researchers have reported that the Bst polymerase in LAMP is less sensitive to the presence of inhibitors than the Taq polymerase used in classic PCR [25], [26]. Our results showed that the LAMP assay is more accurate and sensitive than qPCR methods using simulated human stool samples, and proved markedly faster than qPCR by at least 20 min, thereby significantly shortening the total assay time.

In conclusion, the LAMP assay was successfully validated in this study for rapidity, sensitivity, specificity, and robustness; thus, this assay may serve as an effective means for screening *P. shigelloides* in clinical samples. We proved that the LAMP assay demonstrated superior performance to qPCR in simulated human stool samples, and may facilitate rapid and reliable diagnosis of *P. shigelloides* infections in basic clinical and field laboratories in rural areas.
